# The end of leadership? Person-group fit as a moderator in the relationship between charismatic leadership and individual outcomes

**DOI:** 10.3389/fpsyg.2025.1615936

**Published:** 2025-07-02

**Authors:** Jee Young Seong, Inju Yang, Doo-Seung Hong

**Affiliations:** ^1^College of Business and Economics, Jeonbuk National University, Jeonju, Republic of Korea; ^2^RMIT University, Melbourne, VIC, Australia; ^3^Department of Sociology, Seoul National University, Seoul, Republic of Korea

**Keywords:** charismatic leadership, person-group fit, organizational citizen behavior, task performance, value fit, demands-abilities (DA) fit

## Abstract

**Introduction:**

This study examines charismatic leadership (CL) and its boundary conditions, focusing on their impact on task performance and providing new insights into a phenomenon that has been largely neglected in leadership literature. It examines how person-group (PG) fit moderates the relationship between CL and task performance through followers’ organizational citizenship behavior (OCB).

**Methods:**

Data was collected using survey methodology from two different sources (136 employees and their supervisors) at two points in time in a public-sector firm in Korea. Hypotheses were tested using hierarchical linear modeling.

**Results and discussion:**

Results showed that CL did not affect task performance via OCB when the employees had a high PG value fit. In contrast, employees’ task performance via OCB was positively related to CL when the employees had a high PG demands-abilities (DA) fit. Departing from the dominant conception of leadership and person-environment (PE) fit, we identify the mediating processes between CL and task performance. Thus, we can advance our understanding of CL’s effects on task performance by observing the mediating role of OCB. This study also explores the boundary conditions (PG value and DA fit) in the relationship between CL and task performance through OCB. By examining the two-way interaction between CL and PG fit, this study provides a comprehensive analysis of CL and its boundary conditions that influence task performance.

## Highlights

- CL does not affect task performance via OCB when employees have a high value fit.
- CL affects task performance via OCB when employees have a high DA fit.
- Mediating processes exist between CL and task performance.
- CL and its boundary conditions lead to task performance.

## Introduction

Numerous studies have demonstrated the positive impact of charismatic leadership (CL) on subordinates (e.g., Bass and Riggio, 2006; Den Hartog et al., 2007). Particularly, CL could be a principal mechanism within a group to result in value congruence (Jung and Avolio, 2000; Klein and House, 1995) and shared identity (Cicero and Pierro, 2007; House et al., 2001).

Although the impact of CL on employees has been discussed in various ways, understanding how CL interacts with other organizational contingencies and consequent outcomes is lagged (Bass and Riggio, 2006; Li et al., 2013). Therefore, our paper aims to consider follower characteristics (e.g., person-group or PG fit) as necessary contingencies for CL (Riggio et al., 2008; Siangchokyoo et al., 2020). Given the importance of leadership contexts, including work dynamics (e.g., Ehrnrooth et al., 2023), explicitly incorporating contingencies would help illustrate the relationships between CL and follower outcomes. This would further help us better understand the reality of organizational leadership by answering questions such as when and how much CL would be adequate.

Various individual characteristics would influence how much and which subordinates a leader affects (Klein and House, 1995). We consider fit or congruency between individual members and group characteristics. We explore how CL affects organizational citizenship behavior (OCB) and, further, task performance in a team when there is a high level of person-group (PG) fit (e.g., value fit and demands-abilities (DA) fit). We are interested in OCB and task performance as outcomes of CL because they are directly related to a team’s productivity, either as extra-role or in-role elements of organizational behaviors (Borman and Motowidlo, 1997). We expect CL’s influence on these subordinates’ behaviors is moderated by PG fit.

Although Research indicates a positive effect of CL on follower behaviors, including OCB and task performance (Banks et al., 2017), the degree to which this occurs depends on various contingencies. We propose that a high PG value fit weakens the relationship between CL and leadership effectiveness, whereas a high PG DA fit strengthens it. By introducing new contingencies (i.e., PG fit) and specifying their underlying logic (Kerr and Jermier, 1978), we may widen the current theoretical spectrum in both CL and substitutes for leadership contexts (e.g., Ma et al., 2020).

The current study adopted an interactionist approach (George and Zhou, 2001; Gilmore et al., 2013; Rose, 1962) to further explore the relationships between CL and OCB as well as task performance. The interactionist approach indicates that the influence of CL on follower behaviors is most accurately understood by examining how CL interacts with follower characteristics as a contextual factor. More specifically, this paper is concerned with the moderating role of explicit situational variables that can substitute, neutralize, or enhance CL’s impact on follower outcomes. By drawing on the substitutes for leadership framework (Kerr and Jermier, 1978), this study broadens the applicability of existing knowledge offered by leadership theory. By doing so, we contribute to the applicability of existing knowledge offered by leadership theory. Our exploration sheds light on the conditions under which CL can be successful, thereby contributing to the literature on contingency leadership or the substitutes for leadership framework (Fiedler, 1966; Kerr and Jermier, 1978). Our investigation into how employees’ PG fit moderates the effect of CL on their outcomes highlights the role of followers in CL, which has yet to be understood (Van Dijk et al., 2021).

## Theory and hypotheses development

### Charismatic leadership and organizational citizenship behaviors

Charismatic leadership (CL) is defined as the relationship between a leader and their followers based on the perception of the leader’s exemplary character (Waldman et al., 2001). Charisma is derived from a leader’s exceptional referent power (Bass, 1985). According to the prior research (e.g., Bass, 1985; Choi, 2006; Conger and Kanungo, 1998), charismatic leadership predicts individual outcomes including job attitudes, OCB and task performance. Upon recognizing a charismatic leader’s outstanding qualities, followers are likely to exhibit complete personal devotion to the leader and their articulated vision (Waldman et al., 2001). A leader’s charisma inspires followers to achieve extraordinary outcomes through joint efforts by cultivating collective missions and values (Bass and Riggio, 2006; Shamir et al., 1993; Waldman et al., 2001).

A charismatic leader influences followers to prioritize their collective interests over self-interest and elevate their collective identity (Cicero and Pierro, 2007; House et al., 2001). Important mechanisms that charismatic leaders use to influence their followers could be to put their collective interests above self-interests and enhance their collective identity (Cicero and Pierro, 2007; Conger et al., 2000; House et al., 2001; Shamir et al., 1993). Group-level value congruence induces an escalated sense of homogeneity among followers (Klein and House, 1995). Identification with a group causes followers to adopt their group’s interests as their own (Brewer and Gardner, 1996). Such collective and shared identity strengthened by CL leads followers to engage in more collaborative and altruistic behaviors, such as organizational citizenship behavior (OCB).

The OCB refers to discretionary, extra-role behavior that underpins the social environment in which task performance occurs. Research indicates that the salience of shared identity could increase OCB (Organ et al., 2006). As discussed above, CL would lead to followers’ adaptation to a collective orientation, which generates enthusiasm for altruistic behaviors toward others within an organization, i.e., OCB (e.g., Bass, 1985; Den Hartog et al., 2007). Therefore, our first hypothesis is:

*Hypothesis 1*: Charismatic leadership is positively related to organizational citizenship behaviors.

### Followers’ PG value fit as a contingency factor: “we are the same”

We suggest that followers’ PG fit could modify the influence of CL on OCB. Since follower-related aspects could be essential contingencies limiting leader influence (Kerr and Jermier, 1978), we build an organizing framework for these relationships based on substitutes for leadership theory and research on follower characteristics (Kelley, 1992; Kerr and Jermier, 1978). From an interactionist perspective, individual behaviors are assumed to be determined by the interaction of person and situational factors (Gilmore et al., 2013; Meyer et al., 2010).

Person-group, or PG, fit is the compatibility between an individual and his/her immediate workgroup (Werbel and Gilliland, 1999). It is based on the idea that many employment positions require interpersonal interactions with group members (Werbel and Johnson, 2001). PG fit is likely to increase this motivational component by improving collegial relationships (Werbel and Johnson, 2001). It has been shown to have a positive link to the quality of work relationships, as well as both in-role and extra-role performance (Kristof-Brown et al., 2005). A group context contains various unique characteristics (e.g., values, goals, and skills; Werbel and Gilliland, 1999) that determine how an individual fits in, and the combination of social and task elements is particularly salient in workgroups (Crawford and LePine, 2013; DeRue and Morgeson, 2007).

As for PG fit, Muchinsky and Monahan (1987) presented two distinct types of fit: supplementary and complementary. Supplementary fit refers to the similarity between a person and the characteristics of their workgroup. Perceived PG fit by followers, i.e., value fit and DA fit, captures complementary and supplementary interaction with CL. In particular, PG value fit in the form of supplementary fit corresponds to the value congruence or similarity-attraction paradigm within a group (Ostroff and Judge, 2007). Since values play a vital role in-group identity (Feldman, 1984), value congruence is the most suitable predictor of important outcomes, including OCB (e.g., Chiang and Hsieh, 2012; Wat and Shaffer, 2005).

High value fit as a contingency factor attenuates CL’s influence on followers’ OCB. Substitutes for leadership theory explains that when employees belong to tight-knit groups with high group cohesiveness, the power of leadership is likely diminished (Kerr and Jermier, 1978). This is because positive relationships within a workgroup allow group members to commit to, and identify with, their group (Van der Vegt and Bunderson, 2005). Value congruence could be a contingency for CL since leaders have less influence on the mutual commitment and transformational qualities of followers in groups where followers hold values and identities congruent with each other (Bass and Riggio, 2006; Li et al., 2013; Shamir et al., 1993). High PG value fit, or value congruence, likely leads to a strong sense of social identity, one of CL’s main mechanisms. That is, while identities are inherently social (Mead, 1934), a follower’s high PG value fit would increase collective identity orientation with a group, which makes CL’s impact on the collective identity of a broader social entity less effective or redundant (Howell and Shamir, 2005).

While a certain amount of incongruence between individuals and the organization may be motivating (Argyris, 1964), excessive value fit or conformity in values could promote not simply harmony but also sticking to the status quo or social loafing, both of which negatively affect creativity (Runco, 2004). Following this logic, we propose that PG value fit acts as a substitute for CL and, thus, attenuates the relationship between CL and subordinate OCB. Therefore, we propose the following:

*Hypothesis 2a*: The follower’s PG value fit moderates the relationship between charismatic leadership and organizational citizenship behaviors such that the association is weaker when the PG value fit is high than when it is low.

### Followers’ PG demands-abilities (DA) fit as a contingency factor: “the group needs my ability”

As one of the components of person-job (PJ) fit, DA fit refers to the degree to which a person’s knowledge, skills, and abilities (KSAs) align with the job’s requirements (Cable and DeRue, 2002). This is a complementary fit, as DA fit is achieved when an individual’s characteristics fill gaps that others cannot address (Muchinsky and Monahan, 1987). Traditional job analysis serves as the basis for assessing this fit (Werbel and Johnson, 2001), and this fit can provide salient cues used in developing job-related attitudes and informing work-related decisions (Boon and Biron, 2016). Regarding followers’ PG DA fit as a contingency factor, we expect that high demand abilities (DA) fit further strengthens the positive influence of CL on followers’ OCB.

Individuals who perceive their abilities as closely matching their environment (i.e., high DA fit) focus less on their deficiencies and feel a high level of self-efficacy (Greguras and Diefendorff, 2009). The scarcity of attributes in a group also promotes the activation of independence or unique characteristics (cf. Luria et al., 2019). Similarly, Research shows that employees who work independently are less inclined to share mental models with coworkers (DeChurch and Mesmer-Magnus, 2010) and are less drawn to the collective (Hoffman et al., 2011).

Research suggests CL stimulates group identification by emphasizing shared values in a highly diverse group. In a group with more fragile team identification, team leaders’ devotion to a group identity will significantly affect followers’ attitudes and behaviors, including OCB (Li et al., 2013). While DA fit has little effect on employee attitude (Kristof-Brown et al., 2005), CL can be an intense situation (Mischel, 1977), which provides a clue as to desired specific behaviors. Accordingly, we propose the following:

*Hypothesis 2b*: The follower’s PG demands-abilities fit moderates the relationship between charismatic leadership and organizational citizenship behaviors such that the association is stronger when the PG demands-abilities fit is high than when it is low.

### Task performance via OCB in consideration of PG fit

We expect that CL enhances task performance via OCB. Individual task performance, as reflected in organizational reward systems, is directly related to the organizational and technical core (Borman and Motowidlo, 1997). An individual’s performance at work consists of task performance, and empirical findings indicate a strong positive correlation between OCB and task performance (e.g., Williams and Anderson, 1991). This is because citizenship behaviors underline the maintenance and enhancement of the social and psychological context that supports task performance (Organ et al., 2006).

Some scholars, however, argue that when time is controlled, the relationship between OCB and task performance becomes zero-sum or even a negative correlation, as found in both lab and field studies (Allen and Rush, 1998). Since OCB involves an individual’s active engagement and extra effort (Gilmore et al., 2013), a trade-off between OCB and task performance is expected. Engaging in OCB could harm individual outcomes (Bergeron, 2007). A resource allocation perspective supports this line of argument, as multiple demands must compete for resources between OCB and task performance. Engaging in OCB can be highly strenuous when task demands stretch group members to their limits, requiring adequate attentional resources (Kanfer and Ackerman, 1989).

On the other hand, CL is suggested to be immensely successful in enhancing followers’ performance in situations that followers perceive as demanding (e.g., Bass, 1985; Waldman et al., 2001). CL’s Warmth and trust make followers feel optimistic about acting in the group’s best interest, even in stressful situations (e.g., Tepper et al., 2018). Similarly, a study indicates that CL reduces follower’s strain levels, further facilitating OCB (Boerner et al., 2008). Additionally, since CL is positively related to workgroup identification, followers are more likely to exert extra effort, resulting in higher job involvement and improved group task performance (Cicero and Pierro, 2007; Le Blanc et al., 2021). Therefore, we propose as follows:

*Hypothesis 3*: Organizational citizenship behaviors mediate the relationship between charismatic leadership and task performance.

Furthermore, when considering PG value fit as a contingency factor, CL would be less effective on task performance via OCB when the value fit is high compared to when the value fit is low. The results of the meta-analysis suggest that task cohesion is more closely associated with performance than social cohesion (Beal et al., 2003). A high PG value fit is likely to generate high social cohesion. Employees with similar mental models may be prevented from thinking divergently and acting creatively (Seong and Choi, 2019, 2023), further hindering task performance. Since a high PG value fit attenuates CL’s influence on OCB, as discussed earlier, we propose:

*Hypothesis 4a*: The relationship between charismatic leadership and task performance is moderated by PG value fit through OCB’s mediating effects, so the positive relationship is weaker when PG value fit is high.

It has been addressed that PG DA fit significantly impacts task performance (Kristof-Brown et al., 2005; Seong and Kristof-Brown, 2012). In considering PG DA fit as a contingency factor, CL would positively relate to task performance via OCB when the fit is high rather than low. Our final hypothesis is the following:

*Hypothesis 4b*: The relationship between charismatic leadership and task performance is moderated by PG DA fit through OCB’s mediating effects, such that the positive relationship is stronger when PG DA fit is high.

## Methods

### Data and sample

Data for this Research was gathered from a Korean firm in the public sector. The survey was conducted online in a two-stage process. In Stage 1, team members responded to the questionnaires. In Stage 2, one month after the Stage 1 survey of team members was completed, the team leaders filled out two types of questionnaires: First, to assess the team under their supervision and then, each team member’s performance. By this, we collected the questionnaires from 301 team members. However, among these, only 136 cases were selected because of the non-responses from some leaders to the OCB and the performance of their team members. The team members’ mean age was 41.6 years (SD = 8.2).

#### Charismatic leadership

We developed a four-item scale to assess charismatic leadership, drawing on the work of Waldman et al. (2001). The following are examples: “Our team leaders have our complete confidence in them” and “Our team leader generates respect.” Responses were measured on a seven-point Likert-type scale ranging from 1 (strongly disagree) to 7 (strongly agree) (*α* = 0.98).

#### Person-group value fit and person-group demands-abilities fit

Adopting the items used by Cable and DeRue (2002), we constructed a three-item measure (α = 0.98) to assess the perceived degree of congruence between individuals and their team members. The items used to measure value fit include, “The value of my life is very similar to the value of my team members.” Person-group demands-abilities fit was also measured using three items from Cable and DeRue (2002). A sample item includes, “I feel important to this company because my skills and abilities differ from those that my coworkers possess.” The two variables were also measured on a seven-point scale ranging from 1 (strongly disagree) to 7 (strongly agree) (α = 0.93).

#### Organizational citizenship behavior (OCB)

Ratings of OCB were assessed from team leaders’ responses using Williams and Anderson’s (1991) scales. A sample item is, “Our team members help each other out if someone falls behind in his/her work.” The scales range from 1 (strongly disagree) to 7 (strongly agree) (ɑ = 0.93).

#### Task performance

Team leaders measured task performance using three items adapted from Williams and Anderson (1991). Team leaders responded to items such as “This team achieves its goals” (ɑ = 0.78).

#### Control variables

We controlled several variables that are presumed to predict OCB and task performance. Age and gender were used as control variables in the analyses because they influence the relationship between PG fit and individual-level outcomes (Seong and Choi, 2019). The controls used include age (in years) and gender (male = 1, female = 2). Age distribution by gender is shown in Table 1.

**Table 1:** Age distribution by gender unit: % (*N*).

Age	Male	Female	Total (*N*)
20–29	23.8	76.2	100.0 (21)
30–39	80.0	20.0	100.0 (85)
40–49	98.6	1.4	100.0 (140)
50 and over	100.0	0	100.0 (55)
All	88.4	11.6	100.0 (301)

## Results

We first performed confirmatory factor analyses to examine the distinctiveness of our scales for CL, PG value fit, PG DA fit, OCB, and task performance using AMOS 23.0. We compared this five-factor model with plausible alternative models. Overall, these results demonstrate that the expected five-factor model provides a substantially improved fit over the relevant alternative models [*χ*^2^ (*df* = 109) = 203.91, *p* < 0.001; comparative fit index (CFI) = 0.97, Tucker–Lewis Index (TLI) = 0.97, RMSEA = 0.072, SRMR = 0.048]. Convergent validity was assessed using composite reliability (CR) and average variance extracted (AVE), in accordance with the criteria proposed by Fornell and Larcker (1981). The CR values for all constructs exceeded the recommended threshold of 0.70, and the AVE values were all above the cut-off value of 0.50, indicating satisfactory convergent validity.

Table 2 presents the means, standard deviations, and correlations for the study variables.

**Table 2:** Means, standard deviations, and correlations among study variables^a^.

	Variables	*M*	*SD*	1	2	3	4	5	6	7
1.	Age	41.59	8.16	–						
2.	Gender	1.13	0.34	−0.53^**^	–					
3.	Charismatic leadership	5.36	1.65	0.22^**^	−0.01	(0.98)				
4.	PG value fit	5.65	1.31	0.33^**^	−0.13	0.64^**^	(0.98)		–	
5.	PG DA fit	5.64	1.22	0.41^**^	−0.21^**^	0.35^**^	0.60^**^	(0.93)		
6.	OCB	6.47	0.78	−0.04	0.13	0.26^**^	0.12	−0.15	(0.93)	
7.	Task performance	6.60	0.51	0.02	0.10	0.23^**^	0.16^*^	0.01	0.59^**^	(0.78)

^a^*n* = 136. The alpha internal-consistency reliability coefficients appear in parentheses along the main diagonal.

PG fit, person-group fit; DA fit, demands-abilities fit. ^*^p < 0.05, ^**^p < 0.01.

Key study variables were measured from different sources (i.e., CL, PG value fit, and DA fit by team members, and OCB and task performance by team leaders) to remove the leaders’ effect, and we used hierarchical linear modeling (HLM; Raudenbush and Bryk, 2002) version 6.06 with a restricted maximum likelihood estimation method to test hypotheses. Following a procedure for analyzing conditional indirect effects, we obtained bias-corrected bootstrapped confidence intervals using Hayes (2012) PROCESS program. The PROCESS program allowed us to test our moderated mediation by evaluating the indirect impact of charismatic leadership, PG values, and DA fit on task performance, as mediated through OCB. We tested the hypothesized conditional process modeling (moderated indirect effect, H3) and bootstrapped with 5,000 iterations to construct bias-corrected confidence intervals for the significance tests of the indirect effects. Table 3 summarizes the HLM results for testing all the hypotheses simultaneously.

**Table 3:** Hierarchical linear models: individual-level relationships between charismatic leadership, PG DA fit, OCB, and task performance.

Variables	Model 1: OCB			Model 2: task performance			
	Step 1	Step 2	Step 3	Step 1	Step 2	Step 3	Step 4
*Intercept*	6.48(0.09)^***^	6.48(0.09)^***^	6.48(0.09)^***^	6.59(0.05)^***^	6.59(0.05)^***^	6.59(0.05)^***^	6.59(0.05)^***^
Age	−0.00(0.01)	−0.01(0.01)	−0.01(0.01)	0.00(0.01)	0.00(0.01)	−0.00(0.01)	0.00(0.01)
Gender	0.16(0.21)	0.13(0.18)	0.08(0.16)	0.20(0.15)	0.15(0.14)	0.14(0.14)	0.12(0.13)
Main effects							
Charismatic leadership (CL)		0.09^*^(0.04)	0.03(0.03)		0.08^*^(0.03)	0.01(0.04)	−0.00(0.04)
PG value fit			−0.02(0.06)			0.06 (0.05)	0.07 (0.05)
PG DA fit			−0.06(0.05)			−0.03(0.05)	−0.02(0.05)
Interactive effect							
CL × PG value fit			−0.07^*^(0.03)			−0.04^*^(0.02)	−0.02(0.02)
CL × PG DA fit			0.06^*^(0.02)			0.04^+^(0.02)	0.03(0.02)
Mediator							
OCB							0.21^*^(0.08)
tau	0.3236	0.3300	0.3499	0.0801	0.0853	0.0954	0.1008
*ơ* ^2^	0.2945	0.2824	0.2468	0.1557	0.1455	0.1271	0.1179
Peudo *R*^2^ change		Δ.3454	Δ.3587		Δ.0537	Δ.4973	Δ.3716

*n* = 136. ^*^p < 0.05, ^***^p < 0.001, ^+^*p* < 0.10, PG fit, person-group fit; DA fit, demands-abilities fit.

Hypothesis 1 predicted that CL would be related to OCB. In Step 2 of Model 1, we found that CL is positively related to OCB, controlling for age and gender (*γ* = 0.09, *p* < 0.05). The results from our HLM analysis provide initial support for H1.

**Figure 1:**
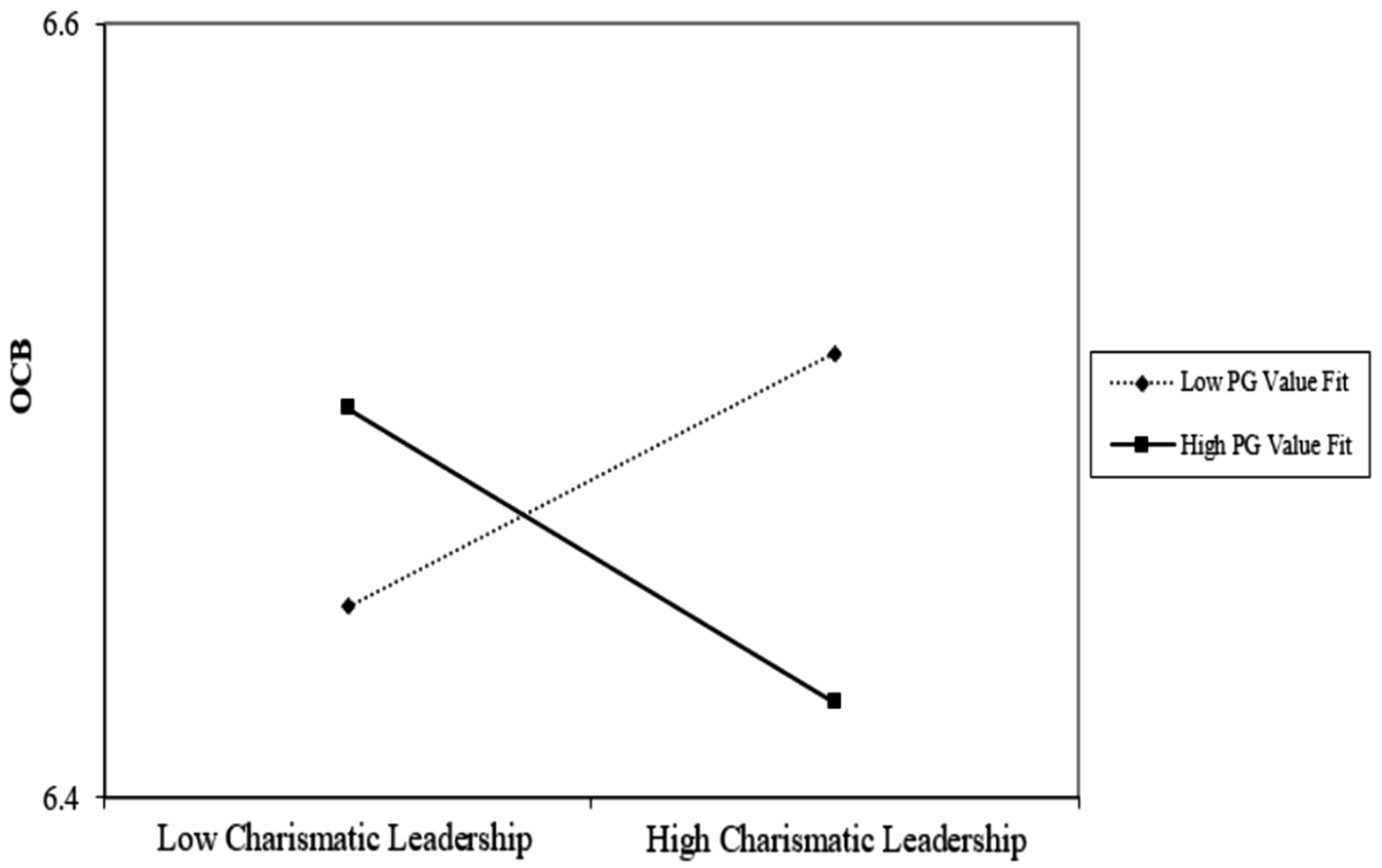
Interaction between charismatic leadership and PG value fit in predicting OCB.

Hypothesis 2a forecasted a significant interaction between CL and PG value fit in predicting OCB. In testing Hypothesis 2a, as shown in Step 3 of Model 1, controlling for age and gender, CL and PG value fit interacted with each other (*γ* = −0.07, *p* < 0.05). Following Aiken and West’s (1991) recommendations, we graphed the interaction effect in Figure 1.

This plot shows the simple effects of charismatic leadership on OCB at high and low levels (±1 SD) of PG value fit. The results of a simple slopes test (Dawson and Richter, 2006) suggested that both at low and high levels of PG value fit, the effect of CL on OCB was statistically insignificant (slope = 0.03, *t* = 1.05; *p* = n.s; slope = −0.04, *t* = −1.28; *p* = n.s. respectively). However, the significant two-way interaction reflects that the slopes of the two lines are substantially different. Thus, PG value fit moderates CL on OCB but in a weak cross-over interaction.

**Figure 2:**
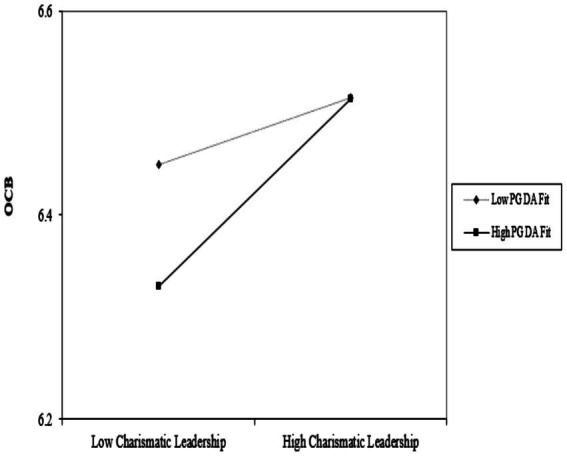
Interaction between charismatic leadership and PG DA fit in predicting OCB.

Hypothesis 2b predicted an interactive effect between CL and PG DA fit that would affect OCB. This interactive effect is also shown in Step 3 of Model 1 (Table 3). Consistent with this hypothesis, there is a significant interactive effect between CL and PG DA fit on OCB (γ = 0.06, *p* < 0.05). Therefore, again, using Aiken and West’s (1991) method, we graphed the interaction effect in Figure 2. This plot shows the simple effects of CL on OCB at high and low levels (±1 SD) of PG DA fit. Simple slope tests showed that the positive slope of CL and OCB was significantly different from zero when PG DA fit was high (slope = 0.09, *t* = 2.03; *p* < 0.05), but at low PG DA fit levels, CL and OCB slope were not statistically different from zero (slope = 0.03, *t* = 1.05; *p* = n.s.). The significant two-way interaction indicates that the slopes of the two lines are substantially different. These results support Hypothesis 2b.

Hypothesis 3 predicted that OCB mediates the relationship between CL and task performance. We followed Baron and Kenny’s (1986) approach to test our hypothesis. Their method of mediation test requires some conditions. The test result for Hypothesis 3 satisfied the first condition of mediation in which the independent variable should be significantly related to the dependent variable (*γ* = 0.08, *p* < 0.05 in Step 2 of Model 2). The second condition is that the independent variable has a significant relationship with the mediator. The test result for the significant relationship between CL and OCB satisfied the second condition (*γ* = 0.09, *p* < 0.05 in Step 2 of Model 1). Finally, the mediator should affect the dependent variable while considering the independent variable simultaneously (Baron and Kenny, 1986). To test the third criterion, we regressed the dependent variable on the mediating variable, controlling CL. As shown in Step 4 of Model 2, OCB was significant (*γ* = 0.21, *p* < 0.05), reducing the coefficient of the effect of CL on task performance (*γ* = −0.00, *p* = n.s. in Step 4 of Model 2). Therefore, the mediation analysis results suggest that OCB fully mediates the effect of CL on task performance.

We then tested the conditional indirect effects of CL on task performance through OCB at different levels of PG value fit and DA fit (Hypotheses 4a and 4b). We employed a bootstrapping procedure to investigate the indirect effect at various levels of moderator variables, including PG value and DA fit. We set a high level of PG fit by adding one standard deviation (SD) to the mean score of PG value fit and a low level by subtracting one SD from the mean (Preacher et al., 2007). However, the indirect effect was not significant. Thus, Hypothesis 4a was not supported.

We expected that the indirect effect of CL on task performance through OCB would become more strongly positive as the degree of PG DA fit moved from a lower to a higher value. We also tested this indirect effect at PG DA fit values equal to the mean plus and minus one standard deviation (Table 4). As expected, the indirect effect of CL on task performance through OCB was conditional upon the level of PG DA fit. The indirect effect was significant and more substantial at a high level of PG DA fit (*b* = 0.0898, bias-corrected bootstrap 95% CI [0.0384, 0.1528], excluding zero). At the same time, it was also significant but weaker at a low level of PG DA fit (*b* = 0.0464, bias-corrected bootstrap 95% CI [0.0151, 0.0841], excluding zero). Thus, Hypothesis 4b was supported.

**Table 4:** Conditional indirect effect(s) of charismatic leadership on task performance at values of PG DA fit.

Path	Moderator	Effect	Boot SE	Boot LLCI	Boot ULCI
Simple path for low PG DA fit	4.67	0.0464	0.0180	0.0151	0.0841
Simple path for high PG DA fit	7.0	0.0898	0.0293	0.0384	0.1528

95% bias-correlated CI.

## Discussion

Our results revealed that CL does not positively affect employees’ task performance via OCB when the employees have a high PG value fit. On the other hand, task performance via OCB is positively related to CL when the employees have a high PG DA fit. We identified the mediating processes between CL and task performance through OCB by examining PG value and DA fit as boundary conditions. This study illustrates that the interactional effects of CL and PG fit may only sometimes further strengthen their positivity together (Sung et al., 2023; Zhang et al., 2025). Specific context could reduce a leader’s influence (Klein and House, 1995), and our paper shows that followers’ PG value fit substitutes and attenuates CL’s effect.

### Theoretical implications

By adopting an interactionist approach and explaining the contingencies of CL, our study extends the boundary conditions for CL (cf. van Knippenberg and van Knippenberg, 2005). Our investigation contributes to the growing body of empirical studies examining the contextual variables that enhance or reduce CL (Ehrnrooth et al., 2023; Kim and Vandenberghe, 2018; Le Blanc et al., 2021; Waldman et al., 2001). Our study is one of the first to investigate how followers’ PG fit impacts the relationship between CL and OCB, and how this relationship, in turn, influences task performance. By considering PG fit as new contingencies, this Research expands our understanding of leadership boundaries (cf. Kerr and Jermier, 1978).

Enhancing either CL or PG fit increases work effectiveness when considered separately. However, a different pattern emerges when we believe their joint factors since increasing both can lead to suboptimal results due to their non-additive configuration. The interactionist approach (Amabile, 1996; Oldham and Cummings, 1996) argues that the leadership process should be examined in terms of how leadership interacts with followers’ characteristics (cf. Gilmore et al., 2013). Our study contributes to Research on followership by investigating followers’ characteristics closely (e.g., Howell and Shamir, 2005; Kelley, 1992; Kim and Vandenberghe, 2018; Klein and House, 1995; Tepper et al., 2018; Van Dijk et al., 2021), beyond the view that a leader is to be the single source of leadership. This study also demonstrates the theoretical and practical value of investigating leader and follower aspects together, rather than in isolation.

Finally, our study contributes to the discussion on PG fit as a contextual variable. Most Research so far has explored fit as either a dependent or independent variable (cf. Vogel and Feldman, 2009). Although modern organizations have widely adopted the application of work systems, relatively little Research has been conducted on person-group (PG) fit, compared to the extensive Research on person-organization (PO) fit (Oh et al., 2014; Seong et al., 2015). Moreover, even those studies regarding PG fit have investigated supplementary fit (in terms of values and goals, e.g., Kristof-Brown et al., 2005) to predict satisfaction with team members, feelings of cohesion, strain, and individual performance. By examining complementary fit (in terms of DA fit), our Research expands our understanding of the dynamics between PE fit and leadership. Simultaneous consideration of the different kinds of fit within a single study would provide a more holistic understanding of an organization and a group (Seong et al., 2015).

### Practical implications

Our findings are particularly pertinent to team-based organizational structures where the role of followers becomes more critical (Li et al., 2013). In such work environments, managers are advised to maximize their OCB and task performance by providing proper CL (Cicero and Pierro, 2007; Conger et al., 2000) and selecting followers with high PG fit (Seong et al., 2015). However, we should note that leadership and followership do not always reinforce one another. Since corporate success is not only a result of leadership per se but also due to effective followers (cf. Kelley, 1992), we must examine how the PG fit of followers interacts with CL of the leaders to influence followers’ receptiveness to leadership.

The results of this paper suggest that charismatic leaders need to recognize that their charisma could be redundant and offer virtually no effect on OCB and task performance in a highly congruent team based on the team members’ values (Kim and Vandenberghe, 2018). The results also suggest that leaders take the pulse of their teams and keep the PG value fit of their followers high. As indicated by our simple slope tests, CL has non-significant or even negative relationships with OCB and task performance when PG value fit is high, particularly in the case of excessive CL.

However, leaders are at an advantage if they exercise CL in situations with high PG DA fit. PG DA fit will help screen out people based on attraction and selection for providing a competitive advantage (Kristof-Brown et al., 2005). In many organizations where job design emphasizes high PG DA fit with clearly designated job responsibilities (Jansen and Kristof-Brown, 2006), CL’s role remains essential for OCB and task performance. A conventional job analysis rigidifying job responsibilities by exclusively focusing on DA fit would help ascertain individual ability to perform the job’s technical aspects. However, since industry moves increasingly toward a more complex and dynamic work environment, a team-oriented work environment requires more flexibility with job responsibilities (Werbel and Johnson, 2001). The presence of leadership becomes vital in fostering team-oriented environments.

Our study supports the argument that influential leaders intervene in subordinates’ affairs to complement and compensate for the latter’s existing abilities and deficiencies (House, 1996). Along with increased diversity in skills and demographic characteristics, CL could benefit the team or the organization in very tangible ways, such as by motivating employees by developing their value congruence and identity. Employees who exhibit a high level of DA fit and job performance are likely to decide to remain in the team or the organization when they feel a greater sense of community and value congruence with the group (Vogel and Feldman, 2009).

### Limitations and future research

From a broader leadership perspective, contingencies may operate in other types of leadership and organizational CL (Grant et al., 2011). Therefore, we suggest that more studies investigate the extent to which different leadership types present either positive (e.g., empowering; Howell and Shamir, 2005) or negative (e.g., abusive or narcissistic) valence in consideration of contingencies (Li et al., 2013; Siangchokyoo et al., 2020).

Given that different types of fit (e.g., person-job, person-group, or person-organization fit) show unique relationships with attitudes and behaviors (Cable and DeRue, 2002), further investigation examining their interactive effects with CL on followers is warranted. Moreover, the supplementary and complementary fit may influence each other over time. For example, individuals who experience a higher value fit are more likely to understand norms and job-specific rules. With less role ambiguity (e.g., Edwards and Cable, 2009), they may invest more effort in acquiring relevant competencies. Over time, they are more likely to be positively reinforced for performing specific tasks (Kristof-Brown et al., 2002).

High leader-member exchange provides employees with better access to the resources necessary to fulfill their job demands (Boon and Biron, 2016). Leadership strengthens the relationship between PG value fit and DA fit perceptions. In addition to Research examining how PG value fit and DA fit affect each other over time (e.g., Boon and Biron, 2016; Shipp and Jansen, 2011), future studies could investigate how they interact with different leadership styles. Longitudinal, experimental, or quasi-experimental designs may help clarify this issue by capturing leaders’ actions across each workgroup’s history (e.g., Meslec et al., 2020; Tepper et al., 2018).

Finally, using the present Korean sample does not necessarily preclude the application of our findings to other cultures. Although the theoretical frameworks and arguments addressed in this article are generalizable across cultures (Den Hartog et al., 1999), certain cultural factors prevalent in Korean culture (e.g., collectivism) may influence the mean scores of specific variables. Relatedly, a meta-analysis of PE fit and work outcomes shows that PG fit has stronger relationships with outcomes in East Asia than in Europe and North America (Oh et al., 2014).

## Conclusion

This study has examined charismatic leadership (CL) and its boundary conditions leading to task performance. It offers new insights into a phenomenon that has been often neglected in leadership literature. It examines how person-group (PG) fit moderates the relationship between CL and task performance through followers’ organizational citizenship behavior (OCB). Departing from the dominant conception of leadership and person-environment (PE) fit, we identified the mediating processes between CL and task performance. Thus, we can advance our understanding of CL’s effects on task performance by observing the mediating role of OCB. This study also explored the boundary conditions (PG value and DA fit) in the relationship between CL and task performance through OCB. Although a significant amount of Research on collective fit has been conducted, a complete account of that mechanism has yet to be given. Additional Research in this area is not only warranted but also critical to advancing our understanding of CL.

## Data Availability

The raw data supporting the conclusions of this article will be made available by the authors, without undue reservation.
